# *“Fear of stopping”* vs *“wanting to get off the medication”*: exploring women’s experiences of using domperidone as a galactagogue - a qualitative study

**DOI:** 10.1186/s13006-021-00438-5

**Published:** 2021-12-09

**Authors:** Gabriella Zizzo, Alice R. Rumbold, Luke E. Grzeskowiak

**Affiliations:** 1 Faculty of Health and Medical Science, The University of Adelaide, Adelaide, Australia; 2 SAHMRI Women and Kids, South Australian Health and Medical Research Institute, Adelaide, Australia; 3 Flinders Health and Medical Research Institute, College of Medicine and Public Health, Flinders University, Adelaide, Australia; 4 SA Pharmacy, SA Health, Adelaide, Australia

**Keywords:** Domperidone, Safety, Safety guidelines, Lactation, Breast milk supply, Galactagogues, Dosage, Tapering, Doctor shopping

## Abstract

**Background:**

Domperidone is the most frequently prescribed medicine used to increase breast milk supply. There is considerable controversy surrounding the use of domperidone in lactation, due to limited evidence about efficacy and concerns about rare but life-threatening side-effects. Despite this, in many high-income settings such as Australia, use of domperidone among breastfeeding mothers appears to be increasing. The aim of this paper was to explore women’s experiences of using domperidone during breastfeeding.

**Methods:**

Semi-structured interviews were conducted in 2019 with 15 women in Australia who reported using domperidone as a galactagogue during breastfeeding. Interviews were recorded, transcribed and analysed thematically.

**Results:**

Women reported a wide variety of practices concerning the timing of initiation of domperidone use, including prophylactic use, as well as the dose and duration of use. Prolonged periods of use and unsupervised dosing were commonly reported, these practices were sometimes associated with a fear of the consequences of stopping, insufficient provision of information about the drug or feeling dismissed by health professionals. Some women indicated that when doctors refused to prescribe domperidone they responded by doctor shopping and seeking anecdotal information about benefits and risks online, leading to unsupervised practices. Women often reported high expectations surrounding the effectiveness of domperidone, and most used the medication in conjunction with food/herbal galactagogues and non-galactagogue support. Positive outcomes following domperidone use included having greater confidence in breastfeeding and pride at achieving breastfeeding goals.

**Conclusions:**

This study identified a variety of practices concerning domperidone use, including potentially unsafe practices, linked in some cases to inconsistent advice from health professionals and a reliance on online, anecdotal information sources. These findings emphasise the urgent need for development of clinical practice guidelines and a greater focus on translating existing evidence concerning domperidone into clinical practice, including clinical support that is tailored to women’s needs.

## Background

The importance of breastfeeding to the mother and baby are well documented. Current and agreed upon advice promotes exclusive breastfeeding until six months and continued breastfeeding (combined with solid food sources) up to two years or longer [1]. Although many studies indicate that women are well-informed about the importance of breastfeeding, the rates indicate a high initiation but low continuation (by six months) in high income countries [2]. Common reasons for breastfeeding cessation in most populations are cited as issues of supply, perceived or real [3–5]. In order to support supply, either to initiate, maintain or boost lactation, women often turn to breastfeeding support and the use of galactagogues which can be certain foods, herbs or medications. Often used as part of traditional practices relied on in many cultures for centuries [6, 7], contemporary galactagogues include dietary and herbal substances including fenugreek, fennel, blessed thistle, goats’ rue, oats and brewer’s yeast, consumed as a tea/tisane, food source or incapsulated as supplements. Although increasing in mainstream use [8] there is currently insufficient evidence supporting the efficacy of galactagogues [4].

The two most commonly prescribed pharmaceutical galactagogues include domperidone and metoclopramide [8]. Both medications are dopamine receptor antagonists that increase prolactin secretion, however, their use during lactation is considered ‘off-label’, with neither medication approved by any regulatory authorities for treating lactation insufficiency [9, 10]. As a result, the use of both medications in this setting has been the subject of much controversy, particularly related to uncertainties regarding their efficacy and concerns about adverse effects [11, 12].

Recent systematic reviews and meta-analyses have demonstrated domperidone to be more effective than a placebo in improving breast milk volume in mothers of preterm infants [13, 14], but evidence remains inconclusive outside of this setting [4]. There also remain uncertainties regarding the population that would most benefit from this treatment. Evidence supporting the efficacy of metoclopramide is less conclusive, while there is some indication that use is associated with a higher prevalence of central nervous system side-effects than domperidone, owing to its ability to cross the blood brain barrier [15]. This has led to domperidone becoming the first-line pharmacological agent of choice for the treatment of lactation insufficiency in many clinical practice settings [16]. Although there is evidence that domperidone increases the corrected QT interval (QTc) and it has been implicated in ventricular arrhythmias and sudden cardiac death, particularly in older and unwell adults, the relevance of these findings to lactating women has been questioned [17].

Despite ongoing controversy regarding the role of domperidone in lactation, recent evidence demonstrates that the prevalence of use has at least doubled over the past decade, particularly in high-income countries such as the UK, Australia and Canada [18–21]. Most notable in these studies is that a large proportion of domperidone use occurs following a full term birth, where evidence supporting its efficacy is less convincing [4, 22].

While there are numerous practice recommendations to support the optimal use of domperidone [9, 10], definite clinical practice guidelines remain largely absent and are reflective of the numerous evidence-practice gaps that exist in the literature. These gaps relate to the optimal dose, when to initiate treatment, duration of treatment, and effectiveness following a term birth and side-effects. In addition, unlike herbal galactagogues, which have been frequently studied [23–27], there has been few studies that have provided an in-depth exploration of the ways in which women are using domperidone during lactation. A recent qualitative study from the Netherlands interviewed 18 mothers and aimed to explore consumer perspectives and experiences regarding the prescribing of domperidone in lactation following the publication of a national clinical practice guidelines [28]. This study identified numerous gaps between what occurred in clinical practice and what the guidelines recommended. Most notable was the haphazard implementation of medical safeguards surrounding domperidone use in lactation. It is unclear whether similar practices occur in Australia.

In light of limited qualitative research, the aim of this paper was to explore women’s experiences of using domperidone during breastfeeding. The research question was “What is the experience of breastfeeding mothers using domperidone to increase breast milk supply?”. The findings will inform healthcare practitioners around the ways in which women use the medications they are prescribed or seek out, with the intention of improving support for lactating women to ensure the risks are understood and medications are being used safely.

## Methods

This study used the interpretive descriptive approach [29] as it facilitates understanding of a phenomenon for the purpose of capturing and interpreting subjective, lived, experiences in a manner which has the ability to inform clinical practice. This analysis represents a sub-study of a larger project exploring women’s awareness, use, and experiences of using substances and other medications for boosting breast milk supply [8]. Data collected from this project have been previously utilised to examine factors that influence women’s decision to use substances to boost breast milk supply [30].

Participants of this study were recruited from a large, online cross-sectional survey conducted in Australia [8]. To attract participants in all states and territories the survey was distributed and shared through various online platforms, including the Australian Breastfeeding Association, the country’s leading breastfeeding advocacy and mother-to-mother support organisation, and Miracle Babies an organisation supporting premature and sick newborns, their families and hospitals. The survey was distributed between September to December 2019. At completion, the survey prompted respondents to express their interest in an interview by providing their name and contact details. A total of 1876 women completed the original survey [8], of which 486 expressed further interest in participating in an interview and provided their contact details. We approached participants in sequential order until we reached the target recruitment number of 20. A total of 46 women were contacted to participate in an interview. Overall, interviews were conducted with 22 women, which included an additional two pilot interviews conducted to test and refine the interview guide. The interviews utilised a semi-structured interview guide, a copy of which has been previously published [30]. GZ conducted all of the interviews. She has a PhD and experience conducting qualitative interviews with mothers around breastfeeding. At the time of the project, GZ was employed as a Research Fellow. Signed consent forms were collected prior to the interviews, which were conducted between October and November 2019. Of the 22 participants, four interviews were conducted in person, two with videoconferencing software and the remaining 16 interviews were conducted over the phone as per the participants preference. These original 22 interviews were used to address the aims of a larger study that investigated factors influencing women’s decision to use substances to boost breast milk supply [30]. Among these 22 participants, 15 indicated that they had used domperidone and their interviews were eligible for inclusion in the analysis to address the aims of this paper. All interviews were audio recorded and provided to a professional transcription service who transcribed them word by word. The first author deidentified transcripts removing names and personal information and assigning each with a pseudonym and the State/Territory they resided in at the time of interview.

The project was approved by the University of Adelaide, Human Research Ethics Committee (H-2019-167). The inclusion criteria for the interviews were intentionally broad and included all women who had previously breastfed or were currently breastfeeding. Only women located in Australia were eligible to participate in interviews. At the completion of the interviews, women were offered an AU$30 gift card to acknowledge their time and contributions.

The semi-structured interviews were conducted by GZ and analysed thematically. The process for thematic analysis was informed by Braun and Clarke’s [31] approach. This approach involved GZ recording detailed memos at the completion of each interview to record key findings including those that contradicted or corroborated findings repeatedly discussed. At the completion of the interviews, the memos and the semi-structured interview guide together with previous literature were used to inform the a priori coding framework. The initial coding framework involved the major topic headings: initiating domperidone, dose, duration of use, and outcomes of domperidone use. The process of familiarisation (multiple and close readings of the transcripts) assisted in refining the coding framework further. The interview transcripts were then coded thematically by GZ using NVivo software (version 12) in multiple cycles and checked by another member of the project team (LEG). Any discrepancies identified were discussed with the third author (ARR). At each cycle the themes were tested and revised further following consultation with the project team. A total of three cycles was necessary to arrive at the final themes.

## Results

### Participant characteristics

Of the 15 interview participants who reported taking domperidone during lactation, ages ranged from 29 to 50 years with babies born between 2009 and 2019. Three of the participants reported delivering a preterm baby (youngest at 27 weeks’ gestation) and nine reported having Caesarean births. The participants reported high levels of completed education with many employed in the healthcare sector or in health-related research. The language predominately spoken by participants in the home was English. One participant was born outside Australia (New Zealand).

Many of the participants reflected on their beliefs about breastfeeding, acknowledging they were not anticipating the initiation and continuation of breastfeeding to be challenging. Those who were aware complications could arise during breastfeeding had either previously experienced breastfeeding and/or supply difficulties or someone close to them had. In general, those who were not anticipating breastfeeding difficulties were not familiar with domperidone before it was introduced to them.

In general, women did not report considering domperidone as a panacea for solving concerns regarding their breast milk supply. They reported taking domperidone in addition to the use of non-pharmacological strategies including obtaining breastfeeding support, more frequent expressing and use of breastfeeding aids (nipple shields, supplementary nursing systems).

Further, in most cases domperidone was used in conjunction with non-galactagogue support and/or other substances such as herbs or lactation cookies. For example, ten participants used domperidone combined with lactation cookies (or other similar products such as bliss balls) that were either homemade, purchased or supplied by friends. Three of the participants took domperidone only and did not use any other galactagogues.

The major themes that emerged within each of the topic areas of; initiating domperidone, dose, duration of use, and outcomes of domperidone use, are outlined below.

### Initiating domperidone

#### Prophylactic versus therapeutic use of domperidone

The timing of the introduction of domperidone varied with some taking it whilst still in hospital post-delivery, and others using it weeks and months after their babies were born. Those who introduced domperidone early (including prophylactically), expected that they would use it briefly to give their supply a ‘kick-start’ as many felt they had missed their milk ‘coming in’ due to circumstances associated with birth i.e., traumatic births, limited to no skin-to-skin contact following birth, no experience of milk coming in or letdown. A small number of women who had previous experiences with low milk supply and expected challenges for subsequent babies, used domperidone prophylactically hopeful that it would thwart supply issues. Others commenced domperidone treatment only after difficulties emerged and after a failed trial of non-pharmacological strategies and/or use of herbal galactagogues.

*So it probably would have been a few days in when my milk still hadn't come in. So of course – well my midwife was more of the natural side of things, so she was – fenugreek and all that sort of – herbs of gold, some of those tablets that already exist sort of thing. Yes. So I went and bought the natural thing and then when it still hadn't come in the domperidone was mentioned as well so I ended up on that. (Bobbi, New South Wales)*

#### Limited prescriber awareness

While women reported that domperidone was most often prescribed by their general practitioner (GP), a number of women reported a general lack of familiarity among prescribers regarding the use of domperidone in lactation, which in some cases led to a reluctance to prescribe domperidone. Even if domperidone was prescribed, some women found the associated advice to be somewhat dismissive:

*What I found unhelpful was that my old GP and then my current GP, they’re the ones that refused the domperidone, and the one who end up giving it to me, were both of the opinion, well he was of the opinion, if I’m not making much milk now, it’s like eight weeks, I should just switch to formula full time. (Margaret, New South Wales)*

Others described interactions with GPs that, despite resulting in a prescription, were often brief and limited in terms of the provision of breastfeeding support and information regarding the optimal use of domperidone. This led to a wide variety of practices, often self-directed by women themselves, with limited treatment supervision. Specifics related to how domperidone was used will be discussed later.

In most cases, women reported presenting to their GP with a direct request for domperidone, based on the advice of another healthcare professional such as a lactation consultant, midwife, or maternal child health nurse. As such, the brevity of information or advice provided by GPs was not initially raised as a concern, as addressing supply needs was seen as the priority.

*And they said if you’re still having trouble, go see your GP. So I went to see a male GP. Just I walked in and I said I want this. And he goes “Do you want like the 25 tablets or 100?”. I said the 100 thanks. (Anita, South Australia)*

#### Inconsistent risk assessments

Women spoke positively about experiences involving health care professionals who were able to provide a comprehensive range of breastfeeding supports as well as prescribe domperidone.

*So, she was a GP, then she became a board certified lactation consultant, and that’s what she does now. So, it was really great, because she could prescribe things, as well as doing the lactation support, so we went into her a bunch of times. (Maria, Western Australia)*

Such interactions with prescribers who provided a more thorough breastfeeding assessment or held a greater interest in lactation, were also more likely to have involved a discussing regarding weighing up the potential risks and benefits of domperidone use, as described by Natalie,

*I wanted to keep breastfeeding, so I don't know, I was just a big dogged about it. I said to my doctor, you know, I don’t take being on medication long-term lightly, and we just talked about it and weighed it up, and I said, I’ve read these things about heart problems, and she said, you know, that’s in a select group of patients, and you don’t have any of those factors, and you know, given my priority on breastfeeding, and obviously the need for [baby’s name] to be fed appropriately, we decided that it was, yeah, okay to be on, long-term. (Natalie, New South Wales)*

Natalie’s experience highlights the process of prescribers screening women for suitability before supplying domperidone, but also the importance of the value placed on breastfeeding by women and the prescribers when undertaking such assessments.

In other cases, women could not recall prescribers ever discussing potential side-effects with them, as described by Karen,

*One of the things I did find really interesting is that nobody talked, well, certainly the GP didn't mention any downsides to using domperidone, as in any potential side-effects. That was quite interesting . . . I went in and said, "I want domperidone," and he kind of went, "Oh okay." I didn't find out for quite a long time that there are actually quite a long list of side-effects. (Karen, Victoria)*

Of note, no women discussed having cardiac monitoring prior to or during domperidone treatment to evaluate or assess potential risks associated with domperidone use.

#### Acceptance of maternal risks

When discussing risks and benefits, there was a clear priority placed on the potential improvement in breast milk supply. Some women emphasised the self-sacrificing nature of decision-making, reflecting moral pressure to breastfeed and that their responsibility to breastfeed was paramount:

*I just felt like I had to – I felt like breastfeeding is the only thing that you’re supposed to do, and I felt like I wanted to feed, so I felt like if I couldn’t breastfeed her, I was failing, and if this medication was a thing that was going to make me be able to breastfeed, then obviously I just needed to do it. (Beth, New South Wales)*

Even in situations where women were aware of potentially serious side-effects, the likelihood of them experiencing such side-effects was most often perceived to be low:

*So I guess part of it is that I have close family friends that are doctors and a midwife. And I talked it through with them as well as well as my actual GP, before I started it. I already knew someone who was taking it. Actually, I knew three people who’d been taking it. And one of them had heart palpitations or she had some heart arrythmia prior to taking it. And even her doctor said ‘oh no it’s fine, we just have to keep an eye on you’. It would explain to me that the risks of taking it are actually not necessarily as dramatic as . . . there’s always a risk but it’s not huge. (Margaret, New South Wales)*

In some cases, women reported having conducted their own thorough risk assessment before requesting domperidone from their doctor:

*He goes “Are you aware there’s side-effects?”. I said “Yes, I’ve done my research”. The side-effects are rare anyway. You know it is something that I suddenly feel passionate about. Breastfeeding was the one thing I could control. Didn’t want to give it up and also I didn’t want to go back to bottles. (Anita, South Australia)*

#### High expectations

Participants reflected on a variety of expectations they had around taking domperidone, but none described having explicit conversations with their prescriber about what to expect when taking domperidone. Many participants assumed domperidone would produce quick results, anticipating high volumes and steady increases, as explained here,

*So it was certainly 24 hours, you know, there was a definite increase there. But it was very temporary and, I mean, I don’t know what I was expecting out of it. I think I thought it would be like a consistent increase. (Karen, Victoria)*

It is unclear where these expectations were derived, but one possible explanation lies in the shared experiences of women who reported having taken domperidone on various social media forums.

### Dose

#### Sense of urgency

Some participants explained that the dose was instructed by doctors, with many starting at a high dose;

*When I was in hospital, my obstetrician, at the time, put me on domperidone, and I think he started that about day four, after my milk hadn’t really come in. Then, we quickly – I think we went straight to the maximum dose, so we went up to the two tablets, three times a day, and I eventually stayed on that for the entire six months, with my daughter. (Alana, South Australia)*

The justification for using high doses to begin with was seen as rational, as Jen explained, she felt a sense of urgency and believed she didn’t have the time required to taper up slowly,

*So, she [the GP] was the one who sort of said “Let’s not – let’s do the domperidone”. So did that, escalated almost instantly. I said “Well, why are we starting at small doses when I’m making nothing?” So, rather quickly we went to max dose. Like, while I did feel a difference, the difference was literally 5mls at max dose. (Jen, New South Wales)*

Unfortunately, in her experience and many others, the higher dose had minimal impact and made little difference to supply.

#### Dose self-adjustment

Others saw the changes to supply as too slow, and with a similar sense of urgency considered the recommended dosages as insufficient, taking it upon themselves to increase their dose unsupervised. As Anita explained, she doubled her dose without the guidance of a doctor or health professional;

*The script was for one tablet three times a day. I noticed a minor improvement but not a lot, so I took it upon myself to double the dose. (Anita, South Australia).*

The way information was delivered or not delivered meant that many women were doing a considerable amount of work collecting information about the best practices from various sources, often with the most trust and authority placed onto information from social media platforms in particular Facebook groups, as explained by Beth:

*Lots of people in the low supply Facebook group seemed to take it [domperidone]. So, most of the information I got, really, was from there. Like, it was there that I heard that other people – and just on social media and stuff – that I heard people took more than they were meant to, and I thought, “oh, I’ll do that too then”. (Beth, New South Wales)*

As Beth explains, this practice of increasing her dose was done without the guidance and support of her doctor and was informed by the anecdotal information she collected online and through social media sources which she deemed to be credible.

### Duration of use

Participants discussed some of the factors that influenced the duration of use including general attitudes about taking medications, fear of stopping and outcomes which varied in terms of intended outcomes such as supply increased, breastfeeding or provision of breast milk was sustained, and at times unintended such as including experiences of side-effects, no change to supply, having to stop breastfeeding.

#### Limited advice and direction

Participants did not explicitly recall conversations they had with prescribers about how long to take domperidone, but as women are often supplied 100 tablets at a time, they were frequently using the entire supply and returning to the original prescriber for repeat prescriptions.

Although many participants discussed long term use of domperidone, in some cases, firstly they were not informed of the possible side-effects of domperidone and secondly, they were not aware or were not warned of any dangers or concerns with the long-term use. This was discussed here by Bobbi,

*But I didn't realise at the time - because I was on domperidone for a while to bring your milk in so no one had told me. I don't know if there's anything wrong staying on it long term actually. I never sort of followed through, but I was probably on it longer than I needed to be for it to be effective anyway. (Bobbi, New South Wales)*

Bobbi did mention that at the time, she may have been informed but due to various other stresses and sleep deprivation, what many called a baby ‘haze’ or ‘fogginess’, she was less able to ask questions or retain information which resulted in her taking domperidone for “a few months”:

*I would probably have dropped the domperidone a lot earlier had I asked the right questions at the right time but I'm not going to beat myself up about that. It's what happened at the time and I probably wasn't in the right frame of mind to be asking. (Bobbi, New South Wales)*

For some women, the stopping and starting of domperidone was sudden, without weaning or tapering off as is commonly recommended. Although many of the women reported they had weaned off domperidone i.e., lowered dosages systematically over a period of time, many indicated that they had not been told to do this or had forgotten. As Anita explained, after extended use, her doctor would not provide any more prescriptions for domperidone and advised her to stop but provided no information about how to do so safely. As she explained, she was not told to taper the dose and instead sourced this information on her own,

*[the GP] said that’s it. You’re not getting more after this so you need to come off. But she didn’t talk to me about weaning. She goes when they’re out, that’s it. I’m not giving you anymore. I knew I had to wean off domperidone through the Facebook group. (Anita, South Australia)*

In the absence of information from her GP, the community she was connected to on Facebook provided Anita with knowledge about the safe practices for coming off domperidone.

For some of the participants, after successfully boosting their supply, they stopped domperidone. However, due to inconsistencies in supply and the ongoing perception of insufficient supply, at each sign of an issue they went back on domperidone. This resulted in multiple periods of taking domperidone on and off for weeks and months. For a number of participants, the episodic use, coupled with the lack of knowledge around safely coming off domperidone indicates that it was often prescribed without follow up or ongoing supervision and with missing information. Thus, the advice around safe usage including tapering advice was not always followed, as confirmed by Alana:

*Well, they haven’t really – no one really ever reviewed it. [Obstetrician] started me on it, then I said to my GP, “oh, my obstetrician started me on this, can you write me another prescription?” She was just like, “yeah, sure”, and that was about it. That’s all the discussion was. Then, it wasn’t anyone saying that I needed to wean off it, I just wanted to get off it myself. (Alana, South Australia)*

#### Fear of stopping

It was not uncommon for participants to continue to use the medication even after an oversupply had occurred, or if there was little to no change in supply, because they were concerned about what effect stopping would have on their milk production. Not wanting to stop the medication was often described or explained as a ‘fear of stopping’ as many viewed domperidone as the only thing sustaining their supply. As illustrated below by Mandy, this fear was reinforced by her lactation consultant’s advice that if it was working not to stop.

*I think I took [domperidone] for about three months . . . Yeah because I was scared to go off it I think more so. I probably could have come off it within a month but I just - it was working for me so much that I just decided to keep going. And I'd get [visiting lactation consultant] back and she'd just say "Well if it's working just keep going". (Mandy, South Australia)*

#### Wanting to get off domperidone

A number of women advised that decisions around the duration of use of domperidone were influenced by their concerns about medications in general or their experiences of possible side-effects.

For participants who experienced side-effects, there was an eagerness to stop domperidone as soon as possible. The women who experienced side-effects described them as including nausea, headaches, lower mood, anxiety and weight gain or generally “making me feel terrible” or “feeling horrible”. No participants reported any cardiac side-effects. For these women the desire to stop domperidone and try alternative options was high.

Despite not experiencing side-effects, there was a small number of women who were keen to stop domperidone quickly as it was their preference not to take prescribed medications, prioritising more natural approaches which they considered safer, as discussed above. As Anita indicated,

*I was keen to come off it. I hate taking tablets . . . I was quite keen to come off of it just because I didn’t want it to be a problem in the future. Like obviously the less medications you have to take, the better [ . . . ] I didn’t want to be on it long term just in case the longer you were on it, the more you would have a risk of being affected. (Anita, South Australia)*

For women like Anita, a reason to avoid long term use was the links they had made between the duration of use and the increasing severity of the side-effects over time. For others, the duration of use was influenced by multiple factors including not wanting to be on medication but also seeing the domperidone as unnecessary because they were not experiencing any change to their supply, as Tammy indicated;

*I think it was like probably pushing a month and then it just didn’t feel like it was working . . . I didn’t want to be on medication the whole time just to breastfeed because I think, subconsciously, I probably thought, in the back of my head, “What is this doing long-term to me and the baby?” (Tammy, South Australia)*

Tammy’s uncertainty here also indicates gaps in the knowledge she had about the medication, particularly around possible long-term effects.

#### Doctor shopping

Some women indicated that doctors were overly cautious in their approach to providing prescriptions for domperidone. Some participants reported that doctors either refused to prescribe domperidone or were not keen to prescribe it for long durations. This resulted in a number of women admitting that they considered or did ‘doctor shop’, the practice of visiting multiple doctors to access domperidone. The GP refusal to prescribe domperidone arose because many felt doctors were not aware or well informed of the practice of “off-label” prescribing or were hesitant to promote behaviours that supported a misuse or overuse of a medication:

*I was on the domperidone and I ended up getting another repeat script but when I went back for the third script, I went to my normal doctor and she said “No, I’m not giving it to you. You need to come off that medication and you either give up breastfeeding or you find other ways to boost your supply”. So frustrated. [ . . . ] That was [by] the third script at 100 tablets. He [son] would have been nearing I think five or six months. (Anita, South Australia).*

As Anita’s experience indicates, not only did women use multiple prescriptions that enabled them to take domperidone for months, there was little evidence that they understood domperidone to be only a short-term treatment. Instead, some viewed the use of domperidone as a long-term treatment (beyond six months) and insisted on going against the doctor’s recommendations saying,

*I didn’t really know anything about [domperidone] until – like I’d never heard of it. Until the nurse told me take it, I’d never heard of it. When I went to the GP, he wasn’t actually that keen to give it me. By the end – this is really bad – by the end, I was doctor shopping so that I could get more because the GP said I couldn’t have anymore. (Beth, New South Wales).*

For participants like Beth doctor shopping was not problematised and was considered a viable solution to address frustrations around doctors’ refusal. In situations where participants were refused medication, we did not hear of them being referred to specialist lactation support as an alternative.

### Outcomes of domperidone use

#### Disappointing results

Several women reflected on their disappointment at taking domperidone and having no or little positive increase in their milk supply. Those who experienced little change to supply resulted in the early cessation of breastfeeding despite a high level of investment and desire to keep going, as discussed by Jen,

*So, then, obviously no supply came, but I mix fed her. It was almost two months by the time we sort of threw in the towel. Alright, well it’s going nowhere. (Jen, New South Wales).*

Some reported that the methods they were using to boost supply, coupled with high levels of anxiety was actually not good for them and had considerable impacts on their mental health and wellbeing.

*Pretty much at six months, it was just sheer exhaustion of, like not being able to exclusively express all the time, and manage everything else that – like, I have to stop (breastfeeding), for my own mental health. (Alana, South Australia)*

#### Empowering outcomes

Some participants indicated that the use of domperidone meant that they could achieve their breastfeeding goals of either direct breastfeeding, long term mixed feeding or exclusive pumping. This was expressed as feeling greater confidence in breastfeeding and/or pride at achieving a milestone or bonding with their baby. The importance of being able to achieve mixed feeding or pumping was significant as a portion of the women reported that although they were not able to sustain breastfeeding, they were able to provide breast milk using these methods, as Bobbi explained;

*But he looked like he sucked so I put him on boob for at least half an hour and not realising he's not doing anything and then I would express for half an hour after that and then I would bottle feed - no. I would feed him on boob, bottle feed him and then express for half an hour and that was every three hours. (Bobbi, New South Wales)*

For a number of women, the pumping and mixed feeding approach coupled with the use of a galactagogue enabled them to produce an oversupply which they stockpiled as explained by Anita:

*But I’m very grateful that there was something that enabled me to continue breastfeeding while I was having trouble because I was pumping after every feed . . . I’ve got a beautiful freezer stash that’s all gone to waste because he’s 12 months old and it hasn’t been touched. But I’d rather have too much milk than not enough (Anita, South Australia)*

Although they were experiencing higher supply than demand, many women continued to use domperidone and non-galactagogues supports to sustain their supply, (possibly associated with their fear of stopping, which they experienced as having a positive effect on their confidence.

## Discussion

The novel findings reported in this study present some previously unreported insight into women’s experiences when using domperidone to support their breast milk supply. In most cases domperidone was used in conjunction with non-pharmacological supports or other galactagogues, suggesting women are not looking to domperidone as a panacea for supporting their breast milk supply. However, this notion is countered by the practice of women using domperidone for long periods of time and often self-adjusting dosages without adequate medical supervision. This was often linked with issues related to low levels of awareness and limited advice and support regarding the use of domperidone among prescribers. Long-durations of use were often accompanied by a ‘fear of stopping’, with women not wanting to take risks involving changes in their breast milk supply. At the same time, we identified inconsistencies in the way that risk-benefit assessments were conducted, while noting a high level of maternal acceptance of potential risks in light of the expected benefits of producing more breast milk. Thus, this research emphasises some key elements that highlight significant evidence and practice gaps regarding the use of domperidone in lactation. The study also provides some important insights into the positive and intended outcomes of using domperidone, highlighting that medications can play a role in supporting women’s long-term breastfeeding goals. The empowering aspects considered alongside the concerning, unsafe and unproven practices suggest that women may require more comprehensive approaches to ensure they are better informed and supported around the safe use of medications to manage breast milk supply. Empowering women around the safe use of domperidone may support them to achieve breastfeeding goals by ensuring they feel heard and are supported in interactions with healthcare providers.

Upon reflection of their breastfeeding expectations, many participants indicated that they did not anticipate the initiations or continuation of breastfeeding to be as difficult as it was. Previous research suggests that when this occurs it is common for women to perceive themselves at fault [32–34] and they are invested in resolving their concerns. Women’s determination to solve breastfeeding or supply issues can foster an ‘at any cost’ response that was seen among some participants in our study, irrespective of potential risks associated with medication use. Other studies have also argued that when challenges arise in women who have a strong determination to breastfed, they often engage in a significant amount of moral “repair work” in order to address what they perceived to be “failures” to breastfeed or supply adequate nourishment for newborn babies [35–37]. Repair work may not always be about aligning with expert advice, but an attempt to align with social expectations around breastfeeding which inform the goals women set for themselves.

The practices of extended use based around a “fear of stopping” and self-directed dose adjustments provides evidence of unsupported/unsupportive practices associated with the use of domperidone. While practices of women initiating both increases in dosage and extension of therapy have been previously noted by Tauritz Bakker et al., [28] reasons for such behaviours have not been well explored. In the context of our study, these practices illustrate how some women navigate the perception of risk and responsibility in the context of breastfeeding challenges, perceiving the risk of baby not having access to breast milk as greater than engaging in unsupported practices that affect their bodies. This implies there may also be an element of sacrifice involved in these practices, Women are not considered to be passive or at fault here as they are either fully cognisant of the risks, the risks are perceived as inconsequential, they have received support that does not align with their goals or does not effectively communicate critical information. This suggests either a communication gap between recommendations/information about galactagogues like domperidone that misinforms practices of use, inconsistency around how the risk is presented or explained, and, a moral burden on women which upholds practices that may be risky to themselves as unimportant vis-à-vis their responsibility to baby. Few women in our study reported active discussions with prescribers regarding potential risks of domperidone use, representing a similar finding to that of Tauritz Bakker et al. [28].

The issues around practices of use that involve self-managed dosing and long-term use was also observed in interviews conducted by Tauritz Bakker et al. with women who took domperidone for lactation insufficiency in the Netherlands [28]. Such practices may be influenced by the lack of agreement by healthcare practitioners around the criteria for use, including no consensus on the safest and optimal dosing [38] and the duration of use. The majority of clinical trials evaluate use over a 7–14-day period, with the longest trialled duration being 28 days [22]. This appears disparate to practice with a previous survey of 1161 domperidone users indicated that fewer than 15% of women used domperidone for less than 28 days, with the majority using it between 1 and 6 months, while 37% used it for six months or longer [15]. This is despite there being no evidence that domperidone leads to improvement in longer-term breastfeeding outcomes, nor evidence that long-term dosing is without potential harms [22]. Nonetheless, women indicated that they are given enough tablets to take for three months up front which may set up an expectation around use. This indicates that evidence and practice are not commensurate and suggests that quantities prescribed may need to be reconsidered. More broadly, these issues indicate that the systems and structures of breastfeeding support, in particular communication techniques and prescribing practices, fosters and enables the unsupported (‘risky’) practices associated with the use of domperidone as a galactagogue and requires urgent attention.

Gaps in communication indicate that women may not be receiving critical information or may be receiving inconsistent information. This results in women needing to rely on multiple sources to inform their practices. Issues around inadequate communication may also reveal a tension between the value women place on advice from experts versus peers or other trusted sources. Although some participants mentioned the value of peer-reviewed evidence as important in their practices and decision making [30] we cannot conclude whether this practice was unique to this cohort as many of them were employed in health or research related fields and therefore had different insights and possibly differing health literacies compared to the general population. However, regardless of education levels and professions, women readily discussed beliefs and engaged in practices that were informed by details garnered on social media or through peers, highlighting a mixed response in terms of where information came from and how women valued and interpreted that information. The discrepancies between what women said they did (valued evidence-based information) and what they actually did (followed anecdotal advice from peers) is not uncommon in health-related research [see [39] who discuss this in relation to obesity reserach] and thus our conclusions can only be speculative. However, we interpret these mixed responses to be a possible consequence of information from professionals being incomplete, missing, inconsistent or conflicting, and women as often problematically dismissed in their interactions with them. The ways in which online forums can be attentive and empathic to women’s emotional, mental, physical, cultural and moral circumstances and privilege women’s concerns and priorities may be why more women view them as informative and supportive spaces [40]. As Lupton [41] indicates, for pregnant and breastfeeding women who readily access online spaces, information is frequently considered more helpful because it is: immediate, regular, detailed, entertaining, customised, practical, professional, reassuring, and seen to be unbiased. In relation to this project, it may well be that women are feeling more heard and validated in online spaces so they come to rely on them as legitimate sources of information representing a shift away from valuing the authority of expert guidance and scientific knowledge that women have readily drawn on in the past [42, 43]. Although women are using online anecdotal resources as a way of assembling and sharing knowledge, which can be validating and empowering, it may also present and promote practices that are unsupported by clinical evidence. Our research gives no indication that women are turning to online sources to *replace* professionals, yet the commensurate value women place on these alternative sources cannot be ignored and instead professionals may need to find ways of incorporating online sources into their support practices.

Issues with communication and misinformation are verified in women’s’ experiences of doctor shopping after being refused the medications and being offered little follow-up support. Whilst the evidence suggests that GPs are applying caution to the prescribing practices, refusal is often not an adequate or appropriate approach to supporting women. Providing women with ultimatums, framed as either-or options such as come off domperidone or give up breastfeeding, is not an approach that is helpful to women as it does not align with and support their breastfeeding goals. Women’s interactions with GPs and prescribing doctors do not seem to indicate whether women were referred to specialists or local breastfeeding support when refused a script. However, women often reported managing information gaps or refusal on their own through self-directed research and problem-solving resulting in doctor shopping and/or unsupported usage. This indicates that GPs may not be promoting breastfeeding support as a first line response and are not taking a shared and collaborative approach to supporting women during this time. This has been identified as an urgent need in existing research that promotes collaboration as a way of improving breastfeeding support [44]. Although women did not consider the pharmaceutical galactagogues a “magic remedy” we are unaware of doctors’ perceptions of the medication and suggest that this may be an area for further exploration.

The findings associated with the continued use of domperidone despite oversupply indicates that for many women, it is not clear what is considered an adequate supply [45]. Women’s view that an oversupply is considered a triumph may be an important indicator that women define an abundance as a characteristic of what is considered sufficient supply. This also indicates that women may be dismissing the importance of a supply-demand connection between their breast milk volumes and over-valuing the concept of stockpiling an excess. The outcome of oversupply may also be deeply linked to the concept of maternal identity and perceptions that an overproduction of breast milk is a way of redeeming or repairing the perceived ‘failure’ typically associated with breastfeeding or supply issues, as discussed previously. From this research it is unclear what is informing these perceptions, but the outcome of producing an oversupply was described by a number of participants indicating women’s expectations and perceptions of breast milk sufficiency may require some further interrogation.

Healthcare professionals that are providing support should not only be aware of the linkages between the strength and determination associated with women’s commitment to breastfeed but also the key decision-making factors women hinge decisions on including the ways they balance safety and risk and how this may inform their decision to tinker with dosages or long-term use [30]. We argue that one way of ensuring more supportive and tailored support that can factor in goals, decision making processes and aspects of maternal identity is a shared decision-making approach as a facet of lactation support delivered by International Board-Certified Lactation Consultants (IBCLC) and other breastfeeding specialists. In addition, GPs may benefit from further training in the best way to support lactating women including the uptake of practice guidelines. This is particularly pertinent given interviews conducted by Tauritz Bakker et al. observed that GPs prescribing domperidone often did so under direction from women themselves and they often relied on the woman’s definition of milk supply problems, rather than assessing the situation themselves [28]. This indicates that more collaborative work needs to be done to ensure women are continually supported whilst ensuring medications are promoted, prescribed and used safely.

### Limitations

Since participants in this study combined galactagogue and non-galactagogue treatments to support breastfeeding or supply challenges this study cannot conclusively report on experiences of the direct effectiveness of domperidone. Further, participants in this study were predominantly highly educated women from English speaking backgrounds. It is possible that the experiences of women from culturally and linguistically diverse backgrounds or those experiencing social disadvantage may be different. Further research that has a specific focus on these populations of breastfeeding women, who often have higher rates of early breastfeeding cessation, is warranted.

## Conclusions

This study provides some key insights into women’s experiences when using domperidone to increase breast milk supply. A wide variety of practices concerning domperidone use are evident, with women often using high dosages for long period of time. Domperidone was often supplied without detailed safety assessments being performed, but when risk-benefits considerations were described, these clearly involved a greater value being placed on the potential benefits of improvements in breast milk supply, rather than possible maternal risks. It was common for women to drive treatment decisions, including dosages and treatment duration, linked in some cases to inconsistent advice from health professionals and a reliance on online, anecdotal information sources. Overall, these findings emphasise the urgent need for development of clinical practice guidelines and a greater focus on translating existing evidence concerning domperidone into clinical practice, including clinical support that is tailored to women’s needs.

## Data Availability

Not applicable.

## Abbreviations

ABA: Australian Breastfeeding Association
GP: General Practitioner
IBCLC: International Board-Certified Lactation Consultant
